# Properties data of phenolic resins synthetized for the impregnation of saturating Kraft paper

**DOI:** 10.1016/j.dib.2018.07.006

**Published:** 2018-07-07

**Authors:** Marion Thébault, Andreas Kandelbauer, Iris Eicher, Björn Geyer, Edith Zikulnig-Rusch

**Affiliations:** a Kompetenzzentrum Holz (Wood K Plus), Altenberger Straße 69, A-4040 Linz, Austria; b Kompetenzzentrum Holz (Wood K Plus), Wood Carinthian Competence Center (W3C), Klagenfurter straße 87-89, 9300 Sankt Veit an der Glan, Austria; c Hochschule Reutlingen, Fakultät Angewandte Chemie, Alteburgstraße 150, D-72762 Reutlingen, Germany

## Abstract

The quality of decorative laminates boards depends on the impregnation process of Kraft papers with a phenolic resin, which constitute the raw materials for the manufacture of the cores of such boards. In the laminates industries, the properties of resins are adapted via their syntheses, usually by mixing phenol and formaldehyde in a batch, where additives, temperature and stirring parameters can be controlled. Therefore, many possibilities of preparation of phenolic resins exist, that leads to different combinations of physico-chemical properties. In this article, the properties data of eight phenolic resins synthetized with different parameters of pH and reaction times at 60 °C and 90 °C are presented: the losses of pH after synthesis and the dynamic viscosities measured after synthesis and once the solid content is adjusted to 45%w/w in methanol. Data acquired by Differential Scanning Calorimetry (DSC) of the resins and Inverse Gas Chromatography (IGC) of cured solids are given as well.

**Specifications Table**

**Table:** 

Subject area	Polymer Applied Science
More specific subject area	Phenolic resin synthesis and properties
Type of data	Tables, Figures.
How data was acquired	pH measurement, Dynamic viscosity, Differential scanning calorimetry (DSC), Inverse Gas Chromatography (IGC).
Data format	Raw and analyzed.
Experimental factors	Eight phenolic resins (P:F = 1:1,8) are synthesized at different pH (Factor A: 8 or 8.5), pre-heating time at 60 °C (Factor B: 0 or 1 h) and reaction time at 90 °C (Factor C: 2 or 3 h).
Experimental features	After synthesis, water is removed by vacuum distillation for the resins that divide into two phases, and all the resins are diluted in methanol to adjust their solid contents to 45%w/w.
Data source location	Kompetenzzentrum Holz (Wood K Plus), Sankt Veit an der Glan, Austria.
Data accessibility	The data are available in this article.
Related research article	These data are supplementary to the article [1].

**Value of the data**

• The data are relating some special conditions of preparation of phenolic resins (resols) with their physico-chemical properties.
• The viscosity profiles of the resins are different before and after dilution in methanol.
• These data can be compared to those of existing resins used in the laminates industry, and can be used as references for the development of resins formulations.
• The data provide information to researchers and industrials that are studying the properties of resols for applied uses, such as the manufacture of laminates.

## 1 Data

Phenolic resins and saturating Kraft papers are the raw materials used in the manufacture of decorative laminates [2], [3], [4], [5]. Papers impregnated with such resins are staked together and pressed at high temperature and pressure [6] to get dense hard boards that can be used as building exterior decoration [7], [8]. The resin and the paper are manufactured to obtain properties that make them suitable for homogenous impregnation, otherwise defects can be found in the boards made thereof [9], [10], [11]. These properties depend on the preparation parameters of these raw materials. The data presented in the present article concern the phenolic resin characterization.

In Table 1 are presented the pH decrease during the resin syntheses, their final solid content and their viscosities.

**Table 1:** Measurement of pH before and after synthesis, solid content and dynamic viscosity for each resin system.

**Resin**	**Initial pH**	**Final pH**	**pH decrease**	**Solid content (%)**	**Viscosity (mPa s)**
1	7.996	7.461	0.535	52.1%	18.8 ± 0.6
a	8.504	7.863	0.641	56.6%	55 ± 1.7
b	8.041	7.485	0.556	51.2%	31.6 ± 1.5
ab	8.505	7.875	0.63	53.2%	24.85 ± 0.85
c	8.027	7.221	0.806	60.3%	54.3 ± 1.2
ac	8.504	7.367*	1.137	76.3%*	2215 ± 175*
bc	7.996	7.127	0.869	72.6%	447.5 ± 39.5
abc	8.514	7.586*	0.928	85.1%*	9800 ± 170*

* After removing the water using a rotary evaporator.

**Fig. 1:**
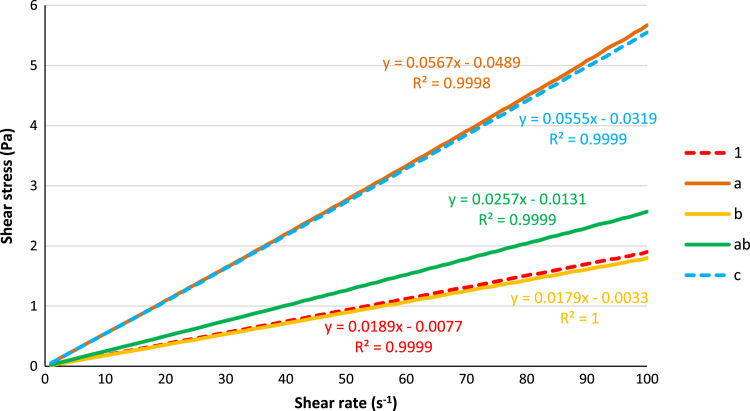
Plots of shear stress with shear rate for the resins 1, a, b, ab and c.

**Fig. 2:**
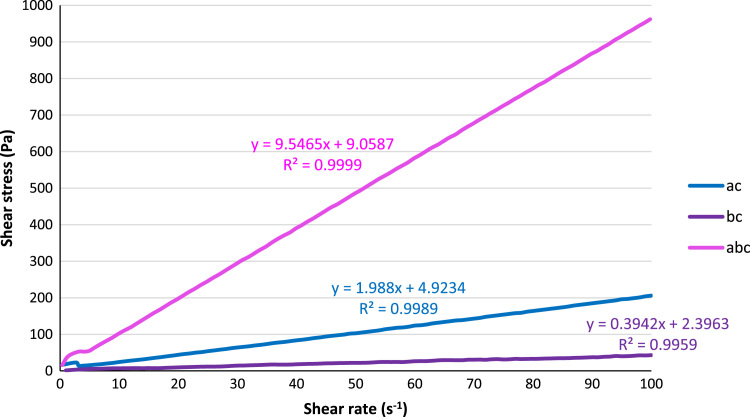
Plots of shear stress with shear rate for the resins ac, bc, and abc.

**Fig. 3:**
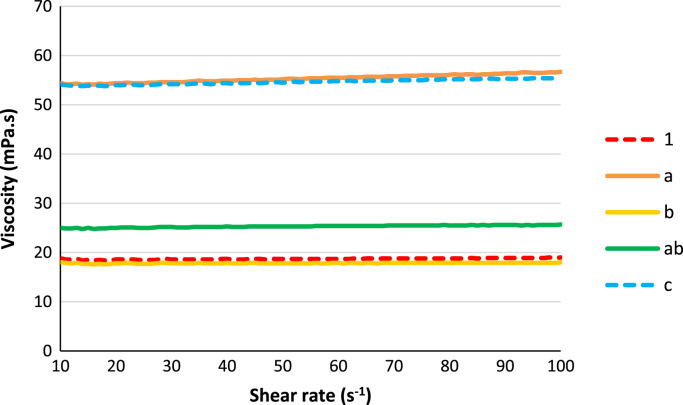
Plots of viscosities with shear rate for the resins 1, a, b, ab and c.

**Fig. 4:**
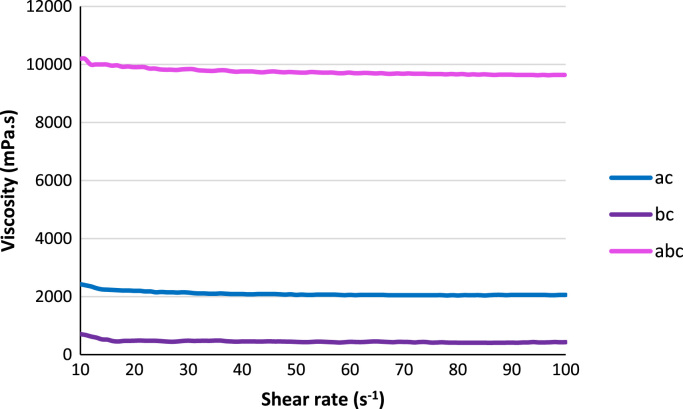
Plots of viscosities with shear rate for the resins ac, bc, and abc.

The following graphs show the plots of shear stress (Fig. 1, Fig. 2) and viscosity (Fig. 3, Fig. 4) with the spindle shear rate of all the resin systems before dilution in methanol.

**Fig. 5:**
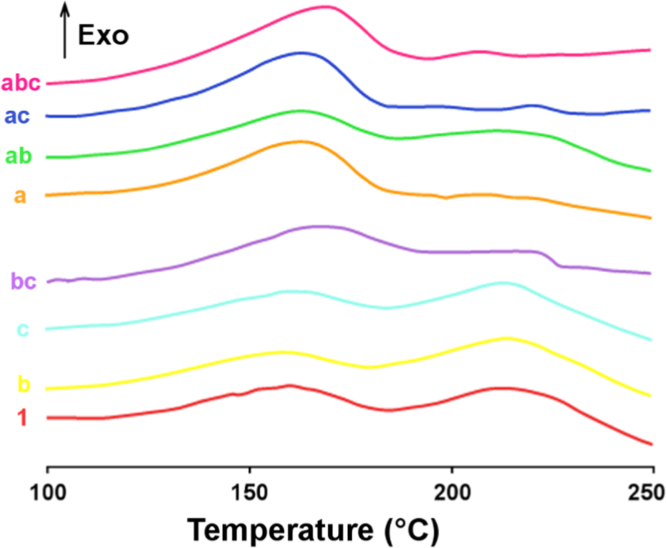
DSC curves of the different phenolic resins.

The resins were characterized by Differential Scanning Calorimetry (DSC). The enthalpy curves are showed on Fig. 5, and the data (onset, peak, endset temperatures and enthalpies) of the detected exothermic peaks in Table 2. These data bring information about the curing properties of the resins.

**Table 2:** Reaction enthalpies (Δ*H*_1_ and Δ*H*_2_), peak temperatures (*T*_*peak*1_ and *T*_*peak*2_), onset temperatures (*T*_o1_ and *T*_o2_) and endset temperatures (*T*_e1_ and *T*_e2_) of the curing of resol resins obtained from the calculations of DSC curves.

**Resin**	***T***_**o1**_**(°C)**	***T***_***peak*1**_**(°C)**	***T***_***e*1**_**(°C)**	**Δ*H***_**1**_**(J/g)**	***T***_**o2**_**(°C)**	***T***_***peak*2**_**(°C)**	***T***_***e*2**_**(°C)**	**Δ*H***_**2**_**(J/g)**
1	121.06	157.49	180.61	− 92.16	198.77	212.66	242.91	− 32.18
a	129.54	163.01	184.20	− 155.15	206.98	229.71	243.53	− 10.61
b	123.83	157.77	177.24	− 48.33	191.07	213.97	240.86	− 77.54
ab	129.34	160.40	182.32	− 91.05	190.43	222.84	239.26	− 27.66
c	124.38	159.09	178.91	− 48.61	191.76	212.13	226.93	− 49.46
ac	131.95	163.15	180.72	− 147.24	210.12	219.86	227.99	− 5.73
bc	136.47	165.17	190.04	− 90.35	204.05	221.29	223.58	− 8.49
abc	130.03	166.74	185.54	− 147.08	197.64	206.5	218.08	− 5.6

In Table 3 are presented the data measured by Inverse Gas Chromatography (IGC) for the cured resin solids. These data bring information about the surface properties of the resins when cured.

**Table 3:** Dispersive surface energies (*ϒ_S_*^D^), specific desorption energies (Δ*G_sp_*) with different standard chemicals, and acid-base constants (*K_a_* and *K_b_*) of cured phenolic resins.

**Resin**	***ϒ***_**S**_^**D**^**(mJ m**^**−2**^**)**	**ΔG**_**SP**_**(mJ m**^**−2**^**)**					***K**_**a**_*	***K**_**b**_*
		**CHCl**_**3**_	**Acetone**	**1,4-dioxane**	**ethylacetate**	**1-butanol**		
1	30,6 ± 1,0	24,9 ± 0,4	42,4 ± 0,8	57,7 ± 0,8	49,1 ± 0,8	49,4 ± 0,9	0,112 ± 0,006	0,192 ± 0,005
a	36,3 ± 2,1	26,8 ± 0,5	46,8 ± 1,3	61,8 ± 1,3	53,7 ± 1,5	52,8 ± 0,8	0,124 ± 0,002	0,201 ± 0,003
b	37,7 ± 2,5	27,4 ± 1,1	48,0 ± 2,6	62,9 ± 2,2	55,1 ± 2,7	53,5 ± 1,6	0,127 ± 0,004	0,203 ± 0,001
ab	33,2 ± 2,5	25,3 ± 1,1	43,8 ± 1,9	59,0 ± 2,1	50,8 ± 2,3	50,3 ± 1,7	0,117 ± 0,007	0,192 ± 0,004
c	34,3 ± 1,2	25,9 ± 0,3	45,0 ± 0,6	60,2 ± 1,0	51,8 ± 0,8	51,7 ± 0,5	0,119 ± 0,005	0,198 ± 0,005
ac	29,9 ± 2,0	24,3 ± 0,9	41,3 ± 1,7	56,9 ± 2,2	48,0 ± 1,9	48,4 ± 1,5	0,110 ± 0,002	0,189 ± 0,002
bc	34,8 ± 1,8	25,7 ± 1,5	45,7 ± 1,7	60,4 ± 2,0	53,0 ± 2,4	51,5 ± 2,7	0,123 ± 0,004	0,191 ± 0,006
abc	40,1 ± 9,6	27,7 ± 3,1	49,8 ± 7,2	63,9 ± 6,2	56,5 ± 7,5	53,8 ± 4,5	0,132 ± 0,005	0,203 ± 0,009

**Fig. 6:**
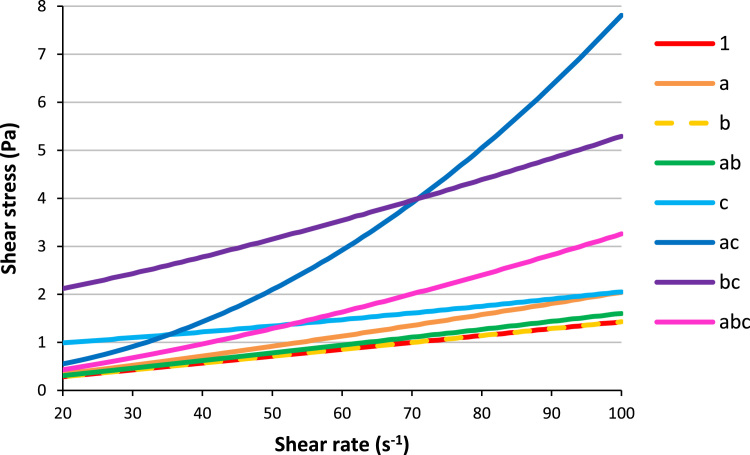
Plots of shear stress with shear rate for all the resins diluted in methanol (45%w/w of solid content).

**Fig. 7:**
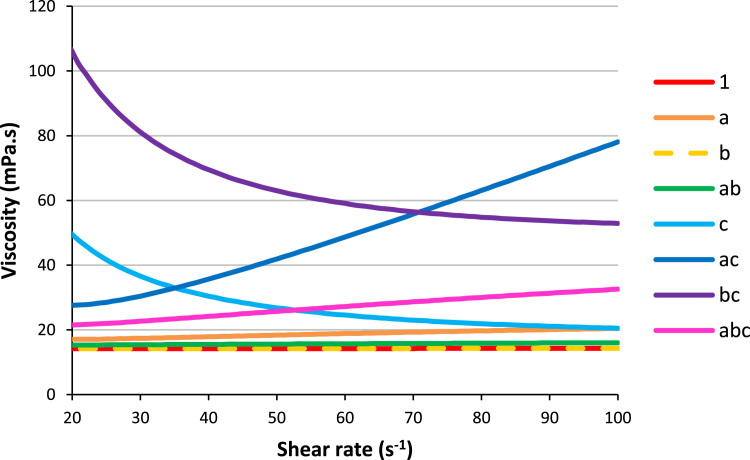
Plots of viscosities with shear rate for all the resins diluted in methanol (45%w/w of solid content) calculated with the Herschel-Bulkley I correlation method.

Before being cured, the resins are often diluted in solvents to be processed. This procedure is often met in decorative laminates manufactures. The following graphs show the plots of shear stress (Fig. 6) and viscosity (Fig. 7) with the spindle shear rate once all the resin are diluted in methanol, adjusting their solid contents to 45% w/w.

## 2 Experimental design, materials, and methods

### 2.1 Resins syntheses

Phenol 99% and formaldehyde solution 37% in water were supplied by Carl Roth (Karlsruhe, Germany). Solid phenol was preheated to melt, and introduced in a 250 ml round-bottom flask. The phenol-formaldehyde molar ratio is P:F = 1:1.8. Sodium hydroxide (catalyst) in pellets was purchased from Sigma Aldrich (Saint-Louis, Missouri, United States). The pellets were dissolved in distilled water to prepare a 45% solution. Different preparations of PF resins were produced by varying the factors pH (factor A, range of pH 8 to 8.5), pre-heating phase (factor B, either none or a pre-heating period of 1 h at 60 °C) and reaction time at 90 °C (factor C, range 2 to 3 h) according to a 2 level-3 factorial experimental screening design (Table 4). The pH of each preparation was measured using a Mettler Toledo Seven Go duo pH-Meter with a precision of 0.001. At the end of each synthesis, the reaction mixture was cooled to room temperature.

**Table 4:** Conditions of preparation of phenolic resins.

**Resin**	**Factor A: pH**	**Factor B: Time of reaction at 60 °C (h)**	**Factor C: Time of reaction at 90 °C (h)**
**1**	8	0	2
**a**	8.5	0	2
**b**	8	1	2
**ab**	8.5	1	2
**c**	8	0	3
**ac**	8.5	0	3
**bc**	8	1	3
**abc**	8.5	1	3

Water is produced during the synthesis, due to condensation of methylolated phenol monomers. For some preparations, where the final liquids tended to separate into two phases (resins ac and abc), vacuum distillation was carried out after the synthesis. The resins were then dissolved in methanol and kept at a maximum temperature of 4 °C until further use.

### 2.2 Viscosity

Dynamic viscosities of the samples were measured with a Physica MCR101 rheometer from Anton Paar, fitted with a conic spindle CP-50-1/01 of 50 mm diameter. The measurements were carried out rotating the spindle from 10 to 100 s^−1^ upon some milliliters of resin sample in a metallic cup at a controlled temperature of 25 °C.

Concerning the resins after synthesis (and remove of water for ac and abc) and before dilution in methanol, the viscosity is calculated according to the relationship:

$$\mu =\frac{\tau }{\frac{d\upsilon }{\mathrm{dy}}}$$

where *μ* is the dynamic viscosity in mPa s, *τ* the measured shear stress in Pa and $\frac{d\upsilon }{\mathrm{dy}}$ the shear rate in s^−1^.

Then the solid contents of all the resins were adjusted to 45% by dilution in methanol. The Herschel-Bulkley I correlation method was used to determine the evolution of viscosity with the shear rate; it was applied in the form *y* = *a* + *b*·*x*^P^.

### 2.3 Differential Scanning Calorimetry (DSC)

All thermograms were recorded with a differential scanning calorimeter 822^e^ DSC equipment by Mettler Toledo (Greifensee, Switzerland). Samples of PF resins of 4 mg were subjected to a temperature gradient ranging from 25 to 250 °C with a heating rate of 10 °C/min. To suppress evaporation of volatiles during condensation, the samples were sealed in high-pressure gold-coated stainless steel crucibles of 30 µl total volume. The enthalpy changes were recorded and analyzed for the peak maximum temperature *T_peak_*, the onset and endset temperatures *T_o_* and *T_e_*, and the normalized enthalpy integral Δ*H*, using the STAR 8.10 software package (Mettler Toledo, Greifensee, Switzerland). All measurements were repeated twice.

### 2.4 Inverse Gas Chromatography (IGC)

Measurements were conducted on an Agilent 6890 gas chromatograph equipped with FID. Chemstation Control Software Version 1.5 (Porotec GmbH, Hofheim/Ts., Deutschland) was used to conduct measurements. Physico-chemical data were evaluated with the analyzing software version 1.1 (Surface Measurement Systems, Alperton Middlesex, London, UK).

Cured solid samples of resins were grind to particles (size less than 1 mm) and filled in silanized glass columns (internal diameter Ø 4 mm). They were conditioned for 4 h under helium gas flow (20 cm^3^ min^−1^) at 35 °C. Samples were measured twice.

Dispersive surface energy $\gamma_{S}^{D}$ was determined using the approach of Schultz et al. [12] injecting n-hexane, n-heptane, n-octane, n-nonane with 0.15 p/p0 at 35 °C. For determination of the specific desorption energies $∆G_{\mathrm{sp}}$ according Schultz et al. [13], chloroform, acetone, 1,4-dioxane, ethylacetate and 1-butanol were used.

Acid-base calculation of *K_a_* and *K_b_* was performed using the approach of Gutman [14].
